# Economic Evaluation of Telerehabilitation: Systematic Literature Review of Cost-Utility Studies

**DOI:** 10.2196/47172

**Published:** 2023-09-05

**Authors:** Sandrine Baffert, Nawale Hadouiri, Cécile Fabron, Floriane Burgy, Aurelia Cassany, Gilles Kemoun

**Affiliations:** 1 Health Economics CEMKA Bourg-la-Reine France; 2 Pôle Rééducation et de Réadaptation CHU de Dijon Dijon France; 3 InterSyndicale Nationale des Internes Paris France; 4 Department of Clinical Research ELSAN Paris France; 5 Centre Clinical Department of Médecine Physique et de Réadaptation Fonctionnelle ELSAN Soyaux France; 6 Laboratoire Mobilité, Mouvement et Exercice (MOVE) – EA 6314 Université de Poitiers Poitiers France

**Keywords:** telerehabilitation, cost-effectiveness, quality-adjusted life year, economic evaluation, cost, rehabilitation, systematic review

## Abstract

**Background:**

Telerehabilitation could benefit a large population by increasing adherence to rehabilitation protocols.

**Objective:**

Our objective was to review and discuss the use of cost-utility approaches in economic evaluations of telerehabilitation interventions.

**Methods:**

A review of the literature on PubMed, Scopus, Centres for Review and Dissemination databases (including the HTA database, the Database of Abstracts of Reviews of Effects, and the NHS Economic Evaluation Database), Cochrane Library, and ClinicalTrials.gov (last search on February 8, 2021) was conducted in accordance with PRISMA (Preferred Reporting Items for Systematic Reviews and Meta-Analyses) guidelines. The inclusion criteria were defined in accordance with the PICOS (population, intervention, comparison, outcomes, and study design) system: the included studies had to evaluate patients in rehabilitation therapy for all diseases and disorders (population) through exercise-based telerehabilitation (intervention) and had to have a control group that received face-to-face rehabilitation (comparison), and these studies had to evaluate effectiveness through gain in quality of life (outcome) and used the design of randomized and controlled clinical studies (study).

**Results:**

We included 11 economic evaluations, of which 6 concerned cardiovascular diseases. Several types of interventions were assessed as telerehabilitation, consisting in monitoring of rehabilitation at home (monitored by physicians) or a rehabilitation program with exercise and an educational intervention at home alone. All studies were based on randomized clinical trials and used a validated health-related quality of life instrument to describe patients’ health states. Four evaluations used the EQ-5D, 1 used the EQ-5D-5L, 2 used the EQ-5D-3L, 3 used the Short-Form Six-Dimension questionnaire, and 1 used the 36-item Short Form survey. The mean quality-adjusted life years gained using telerehabilitation services varied from –0.09 to 0.89. These results were reported in terms of the probability that the intervention was cost-effective at different thresholds for willingness-to-pay values. Most studies showed results about telerehabilitation as dominant (ie, more effective and less costly) together with superiority or noninferiority in outcomes.

**Conclusions:**

There is evidence to support telerehabilitation as a cost-effective intervention for a large population among different disease areas. There is a need for conducting cost-effectiveness studies in countries because the available evidence has limited generalizability in such countries.

**Trial Registration:**

PROSPERO CRD42021248785; https://tinyurl.com/4xurdvwf

## Introduction

### Telerehabilitation

Telerehabilitation refers to the delivery of rehabilitation and habilitation services via a variety of information and communication technologies (ICTs), commonly referred to as “telehealth” technologies. Clinically, the term “telerehabilitation” encompasses a range of rehabilitation and habilitation services that include evaluation, assessment, monitoring, prevention, intervention, supervision, education, consultation, and coaching [1]. This broad definition of Telerehabilitation suggests that the type of ICTs used to support the services is very diverse and is expected to change as technology continues to evolve [2]. Recently, the COVID-19 crisis has increased interest in telerehabilitation and has extended in some countries its perimeter for access and reimbursement [3]. Telerehabilitation is used in several diseases and could benefit a large population in various clinical settings with the aim to improve outcomes by increasing access and adherence to rehabilitation protocols with a positive impact on physical and mental functions and quality of life [4].

### Economic Evaluation

Economic evaluation is a set of formal analytical techniques that provide systematic information about the costs and benefits of alternative therapeutic or preventive options and can thereby assist in decision-making. The objective is to contribute to the efficiency of health care spending and to document value for money to support reimbursement of drugs, medical devices, and activities [5,6]. Many countries have introduced this rationale within their regulations regarding reimbursement and negotiation of the price of innovative new medical products. In France, the economic evaluation of medical products has existed by regulation since 2012 [7], which established the principle of evaluating the efficiency for health products within the framework of the market access process. These evaluations are requested from manufacturers submitting economic evaluations of new medical products (including drugs and devices) that have substantially improved clinical benefits and have a significant impact on budget and an organizational impact on patient management and professional practices. More generally, economic evaluations are performed when assessing public health programs at the national or local level and in the management of health care facilities.

It should be noted that an economic evaluation is only appropriate after its effectiveness and safety have been methodologically soundly demonstrated as a first step. In this respect, the effectiveness of using telerehabilitation has been demonstrated in many studies among different disease areas, and several systematic reviews conclude that telerehabilitation was effective, for example, for patients presenting with musculoskeletal conditions, those with multiple sclerosis, those with impaired mobility [8-12], and those in cardiac telerehabilitation [13]. In the case of pharmaceuticals and devices, the market access dossier of innovations is mainly based on the efficacy and safety results derived from randomized clinical trials. Organizational innovations such as those associated with the use of telerehabilitation raise multiple practical and regulatory issues in the design of interventional studies, which limits their feasibility. In France, as well as in many other jurisdictions including the United Kingdom, Australia, and Nordic countries, the guidelines for manufacturers submitting economic evaluations recommend using cost-effectiveness analysis, where quality-adjusted life years (QALYs) are listed as one of the favored options for measuring effectiveness [14,15]. Over time, QALYs has imposed itself internationally as the gold-standard measure of effectiveness [16,17]. The main reason is the need for consistency in the outcome measures to ensure the usefulness of cost-effectiveness results in decision-making. The existence of a common metric enables the comparison of different kinds of outcomes across disease areas and their comparison with costs in a meaningful way.

### Economic Evaluation of Telerehabilitation

In telerehabilitation, multiple types of clinical outcomes can be considered [18]. QALYs include mortality and morbidity in one single measure that qualifies the years lived weighted by their quality of life. Cost-utility analysis (CUA) involves comparing costs and QALYs. Economic evaluations also consider the dimensions of the cost differential associated with the technology of interest as compared to standard of care defined as the situation of reference. The estimation of costs depends on the perspective chosen from a decision-making standpoint: it is important to clearly define who pays the extra costs or benefits from cost savings. The value of saving money for the society at large or engaging additional resources to support an innovative product or service may be viewed differently by public or private third‑party payers, health providers, governmental agencies, or individual patients.

The extent of cost measurement may then vary deeply in accordance with the scope of the study, suggesting the difficulties and limitations of comparing the results of economic studies performed at an international level and over various time periods. However, even if the transferability of the results of economic evaluation from one setting to another is not straightforward, it remains interesting to benefit from the international experience gained on the economic evaluation of telerehabilitation and especially in focusing on the most ambitious studies based on randomized controlled trials (RCTs) and cost utility.

### Goal of the Study

Despite the existence of 2 systematic literature reviews conducted on cost-effectiveness studies on physical rehabilitation, including telerehabilitation, there is no review about the cost-utility of telerehabilitation to our knowledge [19,20].

The aim of this paper is to review and discuss the use of cost-utility approaches in economic evaluations of telerehabilitation interventions. It is based on a literature review of all published analyses conducted in this field, which used a CUA methodology.

## Methods

This review was planned and conducted in accordance with PRISMA (Preferred Reporting Items for Systematic Reviews and Meta-Analyses) guidelines [21]. It was preregistered on PROSPERO before the search was initiated.

### Inclusion and Exclusion Criteria

The inclusion criteria for this systematic review were defined in accordance with the PICOS (population, intervention, comparison, outcomes, and study design) framework: the included studies had to evaluate patients in rehabilitation therapy for all diseases and disorders (population) through exercise-based telerehabilitation (intervention) and had to have a control group that received face-to-face rehabilitation (comparison), and studies had to have evaluated effectiveness through gain in quality of life (outcomes) and used the design of randomized and controlled clinical studies (study). Studies were included if they met the following criteria: they involved synchronous (real-time and interactive) or asynchronous (store-and-forward) telerehabilitation services with health professionals, they were based on RCTs comparing telerehabilitation with usual in-center rehabilitation, and they reported findings on the cost-utility of telerehabilitation in terms of cost per QALY.

Studies were excluded if they only presented the costs of telerehabilitation. Comments, letters, news articles, editorials, correspondence, narratives, systematic reviews, case studies, study protocols, and articles that were not original or published in non–peer-reviewed journals were also excluded. Finally, when a study was available in different formats or published in several versions, the one containing more information was included. The search has been limited to studies published in French and English until February 8, 2021.

### Literature Search

The following literature databases were used: PubMed, Scopus, Centres for Review and Dissemination databases (including the HTA database, the Database of Abstracts of Reviews of Effects, and the NHS Economic Evaluation Database), the Cochrane Library, and ClinicalTrials.gov. The references of key full-text articles included in the review were checked to identify any potentially eligible studies, including previously published systematic reviews. Search terms were constructed with 2 themes: cost-utility studies and telerehabilitation (Multimedia Appendix 1). Related terms under each theme were combined by using the Boolean operator OR, and the 2 themes were combined using the Boolean operator AND. Additional Boolean operator NOT was used to exclude protocols.

### Study Selection and Data Collection

All identified studies were subject to a 4-step screening process in accordance with the PRISMA framework (identification, screening, eligibility, and included). The search results were exported to an Excel (Microsoft Corp) spreadsheet for exclusion of duplicates. Two independent evaluators assessed the titles and abstracts of relevant studies for inclusion. In case the title abstract did not provide enough information regarding the eligibility criteria, full-text documents were considered. Discrepancies were resolved through discussion until consensus was reached.

The initially selected studies were manually reviewed to identify additional relevant studies. All the references of the articles selected in the first phase were checked for study selection following the same process described previously before the inclusion of the studies.

Two analysts independently extracted data using a common data extraction form.

The following data were extracted for all selected studies: authors, publication year, country of origin, study perspective, pathology of interest, population targeted, sample size, type of intervention, comparator, setting, clinical outcomes studied, time horizon, type of utility data, cost data, economic outcome measure, and authors’ conclusions; QALYs at each time of follow-up, clinical outcomes, and mean differences or standardized mean differences for continuous outcomes with their corresponding confidence intervals; and incremental costs, incremental utility, incremental cost-effectiveness ratio (ICER), and the decision uncertainty is expressed by cost-effectiveness acceptability curves.

Discrepancies in the contents of the full texts of the extracted studies were resolved through discussion.

### Quality Assessment

Two authors independently assessed the methodological quality of the selected studies using the Drummond checklist of the French Health Authority [5,17]. The Drummond checklist was designed to guide the critique of economic evaluations and considers (1) the research question, (2) the description of the study or intervention, (3) the study design, (4) the identification of the cost and consequences of each alternative, (5) measurement, and (6) valuation of costs and consequences, (7) whether discounting was carried out, (8) incremental analysis, (9) presentation of results with uncertainty and sensitivity analyses, and (10) discussion of results in the context of policy relevance and the existing literature.

A component approach was used when applying the checklist in Table 1. This approach is advocated in the PRISMA statement and entails assessing each item individually rather than generating a summary score [22,23].

**Table 1:** Quality assessment of the studies in accordance with the Drummond checklist.

Questions	Studies reporting	Studies, n (%)
The study takes account of both the costs and the outcomes of the intervention.	All studies	11 (100)
The study compares all relevant options on the clinical level.	All studies	11 (100)
A specific viewpoint was adopted, and the study was positioned in a particular decision-making context.	All studies	11 (100)
No important alternative was omitted.	No study	0 (0)
The “do nothing” alternative has been envisaged and studied, if relevant.	N/A^a^	N/A
The alternatives' descriptive elements have been presented (frequency, population analyzed, design of the intervention, etc).	All studies	11 (100)
Effectiveness has been established by a randomized controlled clinical trial, whose protocol reflects what would normally happen in current practice.	All studies	11 (100)
Effectiveness has been established through a summary review of clinical trials of good methodological quality.	N/A	N/A
Effectiveness has been established through observational data or assumptions, with an analysis of biases in the conclusions.	All studies	11 (100)
Have the different relevant viewpoints been examined with regard to costs as well as health effects?	All studies	11 (100)
No important health effect has been omitted. If an important health effect has not been examined, this choice has been justified.	All studies	11 (100)
No important cost has been omitted. If an important cost item has not been examined, this choice has been justified.	All studies	11 (100)
All identified outcomes and cost items have been measured.	All studies	11 (100)
The method used for the quantification of the resources consumed is valid. Unit costs have been detailed (tariffs, market prices, etc) and are suited to the perspective adopted.	All studies except Frederix et al [24]	10 (91)
The measurement of health outcomes is suited to the question posed (life years, event avoided, preference score, etc). The method used to measure the outcomes is valid.	All studies	11 (100)
The sources of information are clearly identified, and the most relevant source has been given priority.	All studies	11 (100)
The costs and outcomes have been discounted at the same rate.	N/A	N/A
The discount rate is known and has been justified.	N/A	N/A
A sensitivity analysis (deterministic and probabilistic) has been presented, covering all uncertain key parameters.	All studies except 3: Frederix et al [24], Frederix et al [25], and Haesum et al [26]	8 (72.7)
In the deterministic analysis, the value intervals have been justified.	Longacre et al [27]	1 (9)
In the probabilistic analysis, the statistical analyses are suited to the nature of the key parameters, and their distribution has been presented and justified.	Longacre et al [27], Kloek et al [28], Fatoye et al [29], Maddison et al [30], Nelson et al [31], and Hwang et al [32]	6 (54.5)
The uncertainty involved in the conclusions of the economic evaluation is known and has been discussed (using CIs, confidence ellipse, or acceptability curve).	Frederix et al [25], Longacre et al [27], Kloek et al [28], Fatoye et al [29], Maddison et al [30], and Hwang et al [32]	6 (54.5)
An analysis of the differences in the costs and health outcomes of the competing alternatives has been conducted and presented.	All studies	11 (100)
If an aggregate indicator has been provided (cost-outcome ratio), it has been correctly interpreted.	All studies except Maddison et al [30] and Nelson et al [31]	9 (81.8)
The alternatives on the cost-effectiveness frontier have been identified.	N/A	N/A
The study is transparent on its limitations.	All studies	11 (100)
The conclusions have been compared, from a critical viewpoint, to those of other studies on the same topic.	All studies	11 (100)
The study addresses the issue of generalizing the conclusions for other contexts or different groups of patients.	All studies	11 (100)

^a^N/A: not applicable.

## Results

### Study Selection

**Figure 1:**
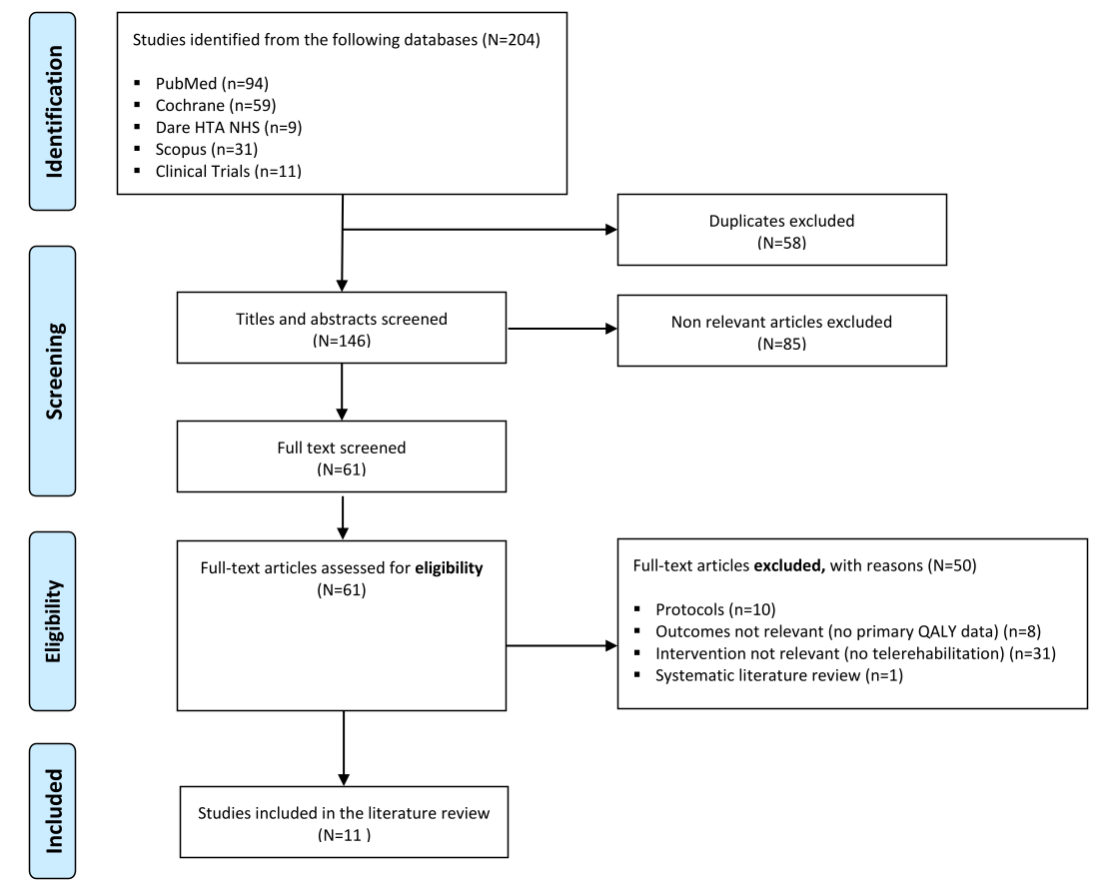
PRISMA (Preferred Reporting Items for Systematic Reviews and Meta-Analyses) flow diagram. Dare: the Database of Abstracts of Reviews of Effects; HTA: the HTA database; NHS: the NHS Economic Evaluation Database.

The search across the aforementioned databases retrieved 204 records. The search across ClinicalTrials.gov retrieved 11 records. After removing duplicates, 146 records remained, of which a further 85 records were excluded as titles and abstracts did not meet the eligibility criteria. During full-text screening, 61 citations were examined in further detail, of which 50 studies were excluded. Finally, a total of 11 economic evaluations were included in the review. The study selection process is shown in Figure 1.

### Study Characteristics

The methodology of the selected studies is summarized in Multimedia Appendix 2 and analyzed in Table 2. Regarding the diseases assessed, 6 concerned cardiovascular diseases, 1 concerned chronic obstructive pulmonary disease (COPD), 1 concerned hip or knee osteoarthritis (or both), 1 concerned patients having undergone total hip replacement, 1 concerned nonspecific chronic low back pain, and 1 concerned cancer. Several types of interventions were assessed as telerehabilitation, consisting in monitoring of rehabilitation at home (monitored by physicians) or a rehabilitation program with exercise and an educational intervention at home alone. All studies met our telerehabilitation criteria with well-specified monitoring frequencies, the use of video for monitoring, and other connected tools. Overall, half of the studies had an intervention duration (usual care and intervention group) of 12 weeks.

**Table 2:** Characteristics of the selected studies (N=11).

Study characteristics				Studies		
				Citations		n (%)
**Region**						
	Europe			Frederix et al [24], Frederix et al [25], Haesum et al [26], Kloek et al [28], Kidholm et al [33], and Kraal et al [34]		6 (55)
	United States and Australia			Longacre et al [27], Maddison et al [30], Nelson et al [31], and Hwang et al [32]		4 (36)
	Africa			Fatoye et al [29]		1 (9)
**Perspective of cost measurement**						
	Societal and health care system			Frederix et al [24], Kloek et al [28], and Kraal et al [34]		3 (27)
	Health care system			Knapp et al [18], Cochrane et al [19], Liu et al [20], Moher et al [21], Frederix et al [25], Longacre et al [27], Kloek et al [28], and Kidholm et al [33]		8 (73)
**Condition**						
	Orthopedics			Kloek et al [28], Fatoye et al [29], and Nelson et al [31]		3 (27)
	Cardiology			Frederix et al [24], Frederix et al [25], Maddison et al [30], Hwang et al [32], Kidholm et al [33], and Kraal et al [34]		6 (55)
	Pulmonology			Haesum et al [26]		1 (9)
	Cancer			Longacre et al [27]		1 (9)
**Sampe size**						
	<100			Fatoye et al [29], Nelson et al [31], Hwang et al [32], and Kraal et al [34]		4 (36)
	100-200			Frederix et al [24], Frederix et al [25], Haesum et al [26], Maddison et al [30], and Kidholm et al [33]		5 (46)
	>200			Longacre et al [27] and Kloek et al [28]		2 (18)
**Time horizon**						
	<1 year			Haesum et al [26], Longacre et al [27], Fatoye et al [29], Maddison et al [30], and Nelson et al [31]		5 (45)
	1-5 years			Frederix et al [24], Frederix et al [25], Kloek et al [28], Kidholm et al [33], Hwang et al [32], and Kraal et al [34]		6 (55)
**Quality of life instruments**						
	SF-6D^a^ or SF-36^b^			Haesum et al [26], Fatoye et al [29], Kidholm et al [33], and Kraal et al [34]		4 (36)
	EQ-5D			Frederix et al [24], Frederix et al [25], Longacre et al [27], Kloek et al [28], Maddison et al [30], Nelson et al [31], and Hwang et al [32]		7 (64)
**Number of utility assessments**						
	2			Frederix et al [25] and Haesum et al [26]		2 (18)
	3			Frederix et al [24], Longacre et al [27], Fatoye et al [29], Maddison et al [30], Nelson et al [31], and Hwang et al [32]		6 (55)
	4			Kidholm et al [33] and Kraal et al [34]		2 (18)
	5			Kloek et al [28]		1 (9)
**Intervention duration**						
	**Usual care group**					
		<12 weeks	Fatoye et al [29] and Nelson et al [31]		2 (18)	
		12 weeks	Frederix et al [24], Kloek et al [28], Maddison et al [30], Hwang et al [32], Kidholm et al [33], and Kraal et al [34]		6 (55)	
		>12 weeks	Frederix et al [25], Haesum et al [26], and Longacre et al [27]		3 (27)	
	**Intervention group**					
		<12 weeks	Fatoye et al [29] and Nelson et al [31]		2 (18)	
		12 weeks	Kloek et al [28], Maddison et al [30], Hwang et al [32], Kidholm et al [33], and Kraal et al [34]		5 (45)	
		>12 weeks	Frederix et al [24], Frederix et al [25], Haesum et al [26], and Longacre et al [27]		4 (36)	

^a^SF-6D: Short-Form Six-Dimension questionnaire.

^b^SF-36: 36-item Short Form survey.

All studies were based on clinical data collected in RCTs. Sample sizes varied from 47 to 516 patients. Only 2 studies had more than 200 participants [27,28].

Four studies had a full societal perspective including health care costs, out-of-pocket patient costs, and productivity loss. Five studies considered only health care costs, 1 included health provider and patient costs, and 1 included only patient intervention costs (Table 1).

All studies carried out a comprehensive cost analysis and included all items of costs relevant to the chosen perspective.

All studies used a validated health-related quality of life (HR-QoL) instrument to describe patients’ health states. Four evaluations used the EQ-5D, 1 used the EQ-5D-5L, 2 used the EQ-5D-3L, 3 used the Short-Form Six-Dimension questionnaire (SF-6D), and 1 used the 36-item Short Form survey (SF-36). No direct valuation method was used to obtain health state utilities. Most evaluations reported the method used to transform the scores from the HR-QoL instrument into utility values. Regarding utility estimates, evaluations in several studies calculated QALYs using the area under the curve method or using the change from baseline score [25-30,33]. In some cases, the calculation was explicitly described [27-30], as for example, the one reported by Longacre et al [27], who calculated QALYs with a conversion of incremental utility gain over the 6-month trial period.

### Quality Assessment

Quality assessment using the Drummond checklist is shown in Table 1. Two reviewers independently conducted the quality assessment for 10% (2/15) of the selected studies. Disagreements were limited to item 6 (“Were costs and consequences valued credibly?”) on the checklist, and examples in Cartwright’s [35] study were consulted to overcome these disagreements. Practical application of item 10 (“Did the presentation and discussion of study results include all issues of concern to the users?”) was challenging due to limited guidance; hence, findings from this question were less informative.

Only 6 studies had a time horizon of 1 year or more. All studies except for those of Haesum et al [26] and Fatoye et al [29] conducted sensitivity analyses on important uncertain variables.

### Evaluation Outcomes

The results of economic evaluations are summarized in Multimedia Appendix 3 and presented in Table 3. The mean QALYs gained using telerehabilitation services varied from –0.09 to 0.89 in the reviewed studies. Nine studies explicitly performed parametric modeling or nonparametric bootstrapping to calculate uncertainty around the costs and effects estimates. These results were reported in terms of the probability that the intervention was cost-effective at different thresholds for willingness-to-pay values. Two studies reported that the QALY gain was not cost-effective [31,34]. Five studies did not report the CI or P values of QALYs [24,27,28,30]. In more than half of the studies, it was not possible to draw any conclusion about cost-effectiveness based on a willingness-to-pay threshold. These studies reported small positive differences in QALYs at increased or similar costs but failed to report significance. All, except for 3 studies [26,29,30] calculated incremental cost per QALY or net monetary benefit.

The main lessons from the 11 studies are that it is dominant (ie, more effective and less expensive) to offer telerehabilitation, which refers to the delivery of rehabilitation and habilitation services via a variety of ICTs used in several diseases.

**Table 3:** Permutation plots summarizing the findings of economic evaluations for interventions versus comparators. Numbers in the cells are number of studies relevant to each permutation.

Incremental costs	Incremental quality-adjusted life years		
	+^a^	0^b^	–^c^
+	1 (Kidholm et al [33])	0	0
0	0	0	0
–	7 (Frederix et al [24], Frederix et al [25], Haesum et al [26], Longacre et al [27], Fatoye et al [29], Hwang et al [32], and Kraal et al [34])	2 (Kloek et al [28] and Maddison et al [30])	1 (Nelson et al [31])

^a^Better health outcomes and higher costs.

^b^Unchanged health outcomes and unchanged costs.

^c^Poorer health outcomes and lower costs.

## Discussion

### Principal Results

This review assesses cost-utility studies of telerehabilitation in comparison with usual care for different diseases and disorders. The general quality of the studies selected in terms of design, statistical methodology, and reporting was quite high. Considering the seminal reviews of telerehabilitation evaluation studies by Bergmo in 2009 [36] and 2014 [37], important progress has been made. However, this may be due to our selection criteria, which were narrower by focusing on telerehabilitation studies based on RCTs.

This review identified 11 economic evaluations with a CUA approach that used QALYs to measure health outcomes. The number of RCTs included in this review might appear quite low compared to the number of studies that use CUA for pharmaceuticals or medical devices.

Most studies originated in northern Europe and Australia, which might be partially explained by extensive expertise in health economics and the request for rigorous evaluations before the widespread adoption of any new health care technology or procedure.

Seven evaluations took the perspective of health providers and intervention costs only and 4 also envisage a societal perspective including costs and benefits for all stakeholders involved.

Most studies showed results about telerehabilitation as dominant, less costly, and with superiority or noninferiority in outcomes. In cases where the incremental utility and ICER were calculated, these values were below the thresholds used in the United Kingdom: the National Institute for Clinical Excellence has recommended that if the ICER is below £20,000-£30,000 (approximately US $25,000-$38,000) per QALY, it is cost-effective.

Results obtained in terms of efficiency based on ICER values or dominant situations provide the expected framework to inform resource allocation by using a common metric, which enables the comparison of different kinds of benefits in multiple disease areas and allows a comparison with costs in a meaningful way. In addition to such a global synthetic presentation of CUA results, it may be noted that detailed intermediate results are also informative in any decision-making process. Disaggregating costs by categories, such as direct or indirect, societal or supported by the health care system, reimbursed or out-of-pocket, provide important information to different stakeholders. The same is true for clinical outcomes, especially to convince clinicians of the benefits of telerehabilitation. According to each therapeutic domain considered in this review, primary clinical end points used to define superiority were diverse.

For patients presenting with cardiovascular disease, Frederix et al [25] calculated the sample size based on a 20% effect size of maximum rate of oxygen consumption attainable during physical exertion (VO_2_ peak), considering a dropout rate of 30% during follow-up. Maddison et al [30] reported that the RCT sample size was based on the assumption of noninferiority in the VO_2_ peak between groups at 12 weeks. In the same type of patients, in 2017, Kraal et al [34] used a physical activity level score, assessed on the basis of physical activity energy expenditure, estimated from an accelerometer and heart rate measured during a period of 5 subsequent days. Conversely, Kidholm et al [33] did not provide any clinical outcome in their study and focused only on the SF-36 instrument as an end point. In patients presenting with heart failure, Hwang et al [32] used the data from a noninferiority trial based on the 6-minute walk distance.

In both studies addressing telerehabilitation for patient populations either after hip or knee replacement or for presurgical patients with osteoarthritis, the primary outcome measure, recorded at 6 weeks, was physical functioning with the Quality of Life subscale of the Hip Disability and Osteoarthritis Outcome Score questionnaire. Despite this common primary end point, conclusions about sample sizes and follow-up periods were contrasted [38].

In the only study focused on patients presenting with advanced cancers [27], the primary clinical outcome was based on a mobility score on the Activity Measure for Post-Acute Care Computer Adaptive Test, measured at different times during follow-up [39].

### Limitations

There are limitations to using QALYs as they might not capture all the benefits of health interventions of interest. Disease-specific HR-QoL instruments are generally more sensitive than generic measures including the EQ-5D or SF-6D in capturing benefits, especially in case of nonsevere conditions [40]. When choosing a utility measure, it is important to consider which instrument is most likely to be sensitive and relevant to changes in health for the specific condition considered. In most studies reviewed, the incremental benefits of QALYs compared to those of standard of care were not statistically significant, which was not surprising considering the limited sample sizes of these RCTs.

One challenge in all economic and clinical evaluations is to balance the need for internal validity against the ability to generalize results to other settings. All studies reviewed were conducted alongside RCTs—a study design associated with specific inclusion criteria for participant inclusion and center selection. Such designs should be discussed for rehabilitation as they may generate bias in the selection of the population enrolled.

Another type of bias, as described in the Cochrane Risk of Bias Tool, is the detection bias resulting from systematic between-group differences in how outcomes are determined [41]. This bias occurs if the knowledge of a patient’s assigned strategy influences the outcome assessment. This situation may occur in RCTs where blinding is not feasible and where patient-reported outcomes, and especially HR-QoL, are considered end points. Patients enrolled in the telerehabilitation arm may be positively influenced by the awareness of benefitting from an innovative process and vice versa for the control.

It is the combination of these results, including those of CUA, as a specific aggregated complement that finally constitute the material of interest for decision-making, letting each stakeholder select the data of primary interest in accordance with their perspective.

### Conclusions

During the last decade, we have underlined important progress in rehabilitation studies, notably with the expansion of the use of innovative technologies. This systematic review suggests that telerehabilitation is a cost-utility approach to improve the accessibility of rehabilitation therapies in a large population in various clinical settings among different areas. This result is important, notably in the recent context of the COVID-19 pandemic, to help determine the appropriate setup for new interfaces for telerehabilitation programs. There were sufficient studies with high levels of evidence on this theme to draw firm conclusions regarding the relative efficiency of telerehabilitation used for several diseases and disorders. There is a need for conducting cost-effectiveness studies in countries because the available evidence has limited generalizability to such countries.

## Appendix

Multimedia Appendix 1 List of search terms.

Multimedia Appendix 2 Studies characteristics.

Multimedia Appendix 3 Studies results.

Multimedia Appendix 4 PRISMA (Preferred Reporting Items for Systematic Reviews and Meta-Analyses) checklist.

## Abbreviations

COPD: chronic obstructive pulmonary disease
CUA: cost-utility analysis
HR-QoL: health-related quality of life
ICER: incremental cost-effectiveness ratio
ICT: information and communication technology
PICOS: population, intervention, comparison, outcomes, and study design
PRISMA: Preferred Reporting Items for Systematic Reviews and Meta-Analyses
QALY: quality-adjusted life year
RCT: randomized controlled trial
SF-36: 36-item Short Form survey
SF-6D: Short-Form Six-Dimension
VO_2_ peak: maximum rate of oxygen consumption attainable during physical exertion
