# Supplementary material for: Economic Evaluation of Telerehabilitation: Systematic Literature Review of Cost-Utility Studies
Source: JMIR Rehabil Assist Technol. 2023 Sep 5;10:e47172. doi: 10.2196/47172 (PMC10509745; doi:10.2196/47172)
Supplement: Multimedia Appendix 1 [file rehab_v10i1e47172_app1.docx]

List of search terms used in database for study selection: telerehabilitation, telehealth, cost effectiveness, cost utility, quality adjusted life years, each searched in title, abstract and keywords. The last search term was protocol searched in title.
