# Supplementary material for: Economic Evaluation of Telerehabilitation: Systematic Literature Review of Cost-Utility Studies
Source: JMIR Rehabil Assist Technol. 2023 Sep 5;10:e47172. doi: 10.2196/47172 (PMC10509745; doi:10.2196/47172)
Supplement: Multimedia Appendix 2 [file rehab_v10i1e47172_app2.docx]

Characteristics of the studies selected

| Author (year) | Country | Perspective of cost measurement | Condition | Target  Population | Sample size | Intervention type | Comparator | Clinical  outcomes | Time horizon | QoL instrument | Utility assessment time |
| --- | --- | --- | --- | --- | --- | --- | --- | --- | --- | --- | --- |
|  |  |  |  |  |  |  |  |  |  |  |  |
| Haesum, (2012)[29] | Denmark | Healthcare system | Chronic obstructive pulmonary disease (COPD) | Patients with severe and very severe COPD | 105 | Telehealth monitor at home for 4 months with data sent to a secured web portal and monitored by physicians that can send recommendations | Home exercise and activities without monitoring by the healthcare professional | admission rate to hospital | 10 months | Convert SF-36 scores into SF-6D | Baseline, 10 months |
| Frederix I, (2015)[32] | Belgium | Societal and Healthcare system | Cardiovascular diseases | Patients with coronary artery disease treated with a percutaneous coronary intervention or with coronary artery bypass grafting, and patients with chronic heart failure with reduced or preserved ejection fraction | 140 | Internet based telerehabilitation**.**  Telerehabilitation with a home motion sensor and a web service. Semi automatic telecoaching, sms and email with tailored recommendations | Centre-based rehabilitation | rehospitalization | 1 year | EQ-5D | Baseline, 6 weeks, 24 weeks |
| Kidholm, (2016)[26] | Denmark | Healthcare system | Cardiovascular diseases (CVD) | Patients with artery sclerosis (myocardial infarction, angina pectoris), coronary artery bypass surgery, valve surgery, and heart failure | 141 | Telehealth monitor.  Healthcare professionals monitored the measured values every week and discussed rehabilitation activities with the patient. | Centre-based rehabilitation | No | 2 years | SF-36 | Baseline, 3 months, 6 months, 12 months |
| Kraal  (2017)[33] | Netherland | Societal and  Healthcare system | Low-to-moderate cardiac risk patients | Patients after an acute coronary syndrome or a revascularisation procedure | 78 | telemonitoring guidance  Telephone (+outpatient clinic) | Centre-based rehabilitation | Physical functioning, Physical activity | 1 year | SF-36 | Baseline, 3 months, 6 months, 12 months |
| Frederix, (2017)[27] | Belgium | Healthcare system | Cardiovascular diseases | Patients of the end of telerehab III (t1) | 126 | Internet based telerehabilitation | Centre-based rehabilitation | cardiovascular readmissions | Up 2years after the intervention | EQ-5D | Baseline, 2 years |
| Hwang (2019) [37] | Australia | Healthcare system | Chronic heart failure (CHF) | Patients with stable CHF | 49 | Telerehabilitation program  exercise and education intervention at home | Centre-based rehabilitation. Education sessions at the hospital on the same day as the exercise sessions and each education session was approximately 60 minutes. | No | 2.5 years | EQ-5D | Baseline, 3 months, 6 months |
| Kloeck JJC  (2018)[24] | Netherland | Societal and Healthcare system | Hip and/or knee osteoarthritis | Patients with hip and/or knee osteoarthritis and not on a waiting list for hip or knee replacement surgery | 208 | Web-application (face-to-face physiotherapy sessions)  face-to-face half-our sessions with a web-application and face-to-face physiotherapy sessions. | Rehabilitation in physiotherapy departments | Physical functioning, Physical activity | 1 year | EQ-5D-3 L | Baseline, 3 months ,6 months 9 months and 12 months |
| Maddison (2019)[30] | Australia | Healthcare system | Coronary heart disease (CHD) | Patients with a CHD within 6 months (atherosclerosis, angina pectoris, myocardial infarction, coronary revascularisation) | 162 | Respoke telerehabilitation platform | Exercise delivered by clinical exercise physiologists in cardiac rehabilitation clinics. Exercise prescription was comparable in both groups. | VO2 max, Physical activity, Body composition, Blood pressure, Blood lipids | 6 months | EQ-5D | Baseline, 3 months, 6 months |
| Nelson  (2019)[34] | Australia | Healthcare system | Total hip replacement | Patients who had received a total hip replacement | 70 | Telehealth monitor (application)  Applications with mobile internet and physiotherapy session via real-time videoconferencing | Traditional care: standardised paper-based home exercise programme. | Time burden | 6months | EQ-5D-5L | Baseline, 6 weeks, 26 weeks |
| Fatoye  (2020)[28] | Nigeria | Healthcare system | Non-specific chronic low back pain (NSCLBP) | Patients with NCLBP | 47 | Application App incorporated personalized and guided self-therapy | McKenzie extension protocol and a set of back care education instructions comprising a 9-item instructional guide on standing, sitting, lifting, and other activities of daily living at home | LBP disability index | 8 weeks | SF-6D (Oswestry Disability Index) | Baseline, 4 weeks, 8 weeks |
| Longacre (2020) [25] | United States | Healthcare system | Advanced cancers | Patients with advanced cancers, pathologically confirmed stage IIIC or IV solid or hematologic cancer and moderate functional impairment | 516  (3 arms) | Telephone centralized tele-rehabilitation +automated  home-based monitoring of physical functioning and pain via telephone and/or internet (3 arms) | Only automated  home-based monitoring of physical functioning and pain via telephone and/or internet | No | 6 months | EQ-5D-3L | Baseline, 3 month, 6 month |
