# Supplementary material for: Economic Evaluation of Telerehabilitation: Systematic Literature Review of Cost-Utility Studies
Source: JMIR Rehabil Assist Technol. 2023 Sep 5;10:e47172. doi: 10.2196/47172 (PMC10509745; doi:10.2196/47172)
Supplement: Multimedia Appendix 3 [file rehab_v10i1e47172_app3.docx]

Study results

| Author, year | Currency | Interventions Comparators | Total (mean) costs | Incremental costs | QALY | Incremental QALY | ICER | Sensitivity analysis |
| --- | --- | --- | --- | --- | --- | --- | --- | --- |
|  |  |  |  |  |  |  |  |  |
| Haesum, 2012 [29] | € | Intervention group | 7 862 | - 288 | 0.013 | 0.027  (NS^b^) | Dominant | Yes ? |
|  |  | Control group | 8 150 |  | -0.014 |  |  |  |
| Frederix, 2015 [32] | € | Intervention group | 2 156 | -564.40 | 0.06 | 0.03  (adjusted)  Statistical significance ?) | Dominant | Yes |
|  |  | Control group | 2 720 |  | -0.09 |  |  |  |
| Kidholm, 2016 [26] | € | Intervention group | 5 724 | 1 667 | 0.089 | 0.004  (NS^b^) | 416 750 €/QALY | Yes |
|  |  | Control group | 4 057 |  | 0.085 |  |  |  |
| Kraal, 2017 [33] | € | Intervention group | 11 772 | - 6 086  (NS^b^) | 0.77 | -0.01  (NS^b^) | Dominant ? | Yes |
|  |  | Control group | 17 858 |  | 0.78 |  |  |  |
| Frederix, 2017 [27] | € | Intervention group | 3 262 | - 878 | 0.07 | 0.22  (Statistical significance ?) | Dominant | Yes |
|  |  | Control group | 4 140 |  | -0.15 |  |  |  |
| Hwang, 2019 [37] | Australian $ | Intervention group | 2 325 | -1 590.45 | 0.36 | NS^b^ | Dominant | Yes |
|  |  | Control group | 3 916 |  | 0.36 |  |  |  |
| Kloeck, 2018a  (societal) [24] | € | Intervention group | 6 348 | -1 .371  (- 529 ajusted) |  | 0.01  (NS^b^) | na^a^ | Yes |
|  |  | Control group | 7 718 |  |  |  |  |  |
| Kloeck, 2018b  (Healthcare system) [24] | € | Intervention group | 3 928 | 1 177  (- 792 adjusted) |  | 0.01  (NS^b^) | na^a^ |  |
|  |  | Control group | 5 105 |  |  |  |  |  |
| Maddison, 2019 [30] | NZ$ | Intervention group | 4 920 | - 4 615  -4 615 | na | na^a^ | na^a^ | Yes (cost) |
|  |  | Control group | 9 535 |  | na |  |  |  |
| Nelson, 2019 [34] | Australian $ | Intervention group | 487 | -28.9 | 0.88 | -0.0025  (NS^b^) | 11 560€/QALY  (NS^b^) | Yes |
|  |  | Control group | 516 |  | 0.89 |  |  |  |
| Fatoye, 2020 [28] | $ | Intervention group | 62 | - 45 | 0.085 | 0.001  (NS^b^) | Dominant | ? |
|  |  | Control group | 106 |  | 0.084 |  |  |  |
| Longacre, 2020 [25] | $ | Intervention group B | 155 |  |  | 0.01 | 15 494$/QALY | Yes |
|  |  | Intervention group C | 270 |  |  | 0.0075 | Dominant |  |
|  |  | Control group A | - |  |  | 0 |  |  |

^a^na : not available

^b^NS :non significant
